# Genetic Divergence and Functional Significance of Bioactive Compounds in Rice and Barley: Implications for Biofortification and Human Health

**DOI:** 10.3390/ijms26157374

**Published:** 2025-07-30

**Authors:** Essam ElShamey, Jiazhen Yang, Xiaomeng Yang, Md. Mahmudul Hasan, Tao Yang, Yawen Zeng

**Affiliations:** 1 Biotechnology and Germplasm Resources Institute, Yunnan Academy of Agricultural Sciences, Kunming 650205, China; yangjiazhen415@163.com (J.Y.); yxm89ccf@126.com (X.Y.); mmhasanum@gmail.com (M.M.H.); yt52279076@163.com (T.Y.); 2 Rice Research Department, Field Crops Research Institute, Agricultural Research Center, Cairo 12619, Egypt

**Keywords:** GABA, resistant starch, alkaloids, rice, barley, QTL mapping, biofortification, health benefits

## Abstract

The functional components in cereals (rice and barley), such as gamma-aminobutyric acid (GABA), resistant starch (RS), and alkaloids, play crucial roles in human health, offering benefits such as improved cardiovascular function, enhanced gut microbiota, and potential anticancer properties. Rice (*Oryza sativa*) and barley (*Hordeum vulgare*) are key dietary staples with distinct genetic architectures influencing the biosynthesis and accumulation of these bioactive compounds. In this study, we explore the interaction and divergence of gene loci associated with GABA, RS, and alkaloid pathways in rice and barley, leveraging comparative genomics to identify conserved and species-specific regulatory mechanisms. We highlight key quantitative trait loci (QTLs) and candidate genes, such as GAD (glutamate decarboxylase) for GABA synthesis, *SSIIa* and *GBSS* for RS formation, and alkaloid biosynthesis genes including *CYP80G2*. Additionally, we discuss the health implications of these functional components, including their roles in reducing hypertension, managing diabetes, and exhibiting neuroprotective effects. Understanding the genetic differences between rice and barley in accumulating these compounds can guide biofortification strategies to enhance nutritional quality in cereal crops, ultimately benefiting human health and dietary outcomes.

## 1. Introduction

Rice (*Oryza sativa*) and barley (*Hordeum vulgare*) are among the most widely consumed cereals, serving as staple foods for billions of people worldwide [1,2]. Beyond their roles as primary energy sources, these grains contain bioactive compounds that contribute to human health, including gamma-aminobutyric acid (GABA), resistant starch (RS), and alkaloids [3]. The interactions among these components influence the nutritional and functional properties of rice and barley, making them valuable in the development of functional foods aimed at preventing chronic diseases and improving metabolic and neurological health [4,5,6]. The human chronic disease outbreak is associated with the change in staple food from brown rice and barley for ancient people to white rice and barley flour for modern people, which divided the six major dietary structures. In fact, sustaining the consumption of major foods plus barley grass powder is the healthiest dietary guideline for modern humans. More than 30 functional ingredients in barley grass exert potent preventive effects on over 20 chronic diseases, and 15 functional ingredients in barley grains prevent 11 chronic diseases [7,8]. In this article, we review the key functional components in both rice and barley, as well as their potential health benefits. GABA, a major inhibitory neurotransmitter in the central nervous system, has been linked to anti-hypertensive, anti-stress, and neuroprotective effects [9,10]. In rice and barley, GABA levels increase during germination, fermentation, and other stress-induced processes, enhancing their functional food potential [11]. Meanwhile, resistant starch, a type of dietary fiber that escapes digestion in the small intestine, acts as a prebiotic, promoting gut microbiota diversity and improving insulin sensitivity [12]. The fermentation of RS by gut bacteria may also influence GABA production, suggesting a potential gut–brain axis interaction [13]. Rice is a rich source of starch [12], which provides energy and acts as a functional ingredient in food processing. RS in rice has prebiotic effects, promoting gut health by feeding beneficial gut bacteria [14]. Brown rice and whole-grain rice contain significant amounts of dietary fiber, which aids digestion, reduces cholesterol levels [15,16], and helps regulate blood sugars [17]. Additionally, rice contains B vitamins (e.g., thiamine and niacin) and minerals such as magnesium, phosphorus, and selenium [18]. These nutrients support metabolism, bone health, and immune function.

Although they are less studied in cereals compared to medicinal plants, alkaloids exhibit bioactive properties, including antioxidant, anti-inflammatory, and neuromodulatory effects [19,20]. Certain alkaloids may interact with GABA receptors or affect starch digestion, further complicating the metabolic interplay among these compounds. The presence and bioactivity of these molecules depend on genetic factors, environmental conditions, and post-harvest processing techniques, such as soaking, germination, and fermentation [21,22]. Understanding the synergistic and antagonistic interactions among GABA, resistant starch, and alkaloids in rice and barley is crucial for optimizing their health benefits. For instance, GABA-enriched germinated brown rice is marketed for its anti-anxiety and cardiovascular benefits, while high-RS barley products are used for diabetes management and gut health promotion. However, the potential modulation of these effects by alkaloids remains underexplored.

Barley is considered one of the richest sources of beta-glucans, which are functional components and a type of soluble fiber. These are known to lower cholesterol, improve heart health, and regulate blood glucose levels [23]. Additionally, barley contains both soluble and insoluble fiber, which aid digestion, promote satiety, and support gut health [24]. Similarly to rice, barley contains resistant starch, which acts as a prebiotic and supports gut microbiota [25]. Alkaloids are a diverse group of naturally occurring nitrogen-containing compounds that have garnered significant attention due to their pharmacological, ecological, and nutritional properties. Regarding which alkaloids are commonly associated with medicinal plants, recent research has highlighted their presence and functional roles in stable crops such as barley (*Hordeum vulgare*) [26]. Functional foods are designed to provide health benefits beyond basic nutrition, often through bioactive compounds such as polyphenols, prebiotics, and probiotics. A key factor in their efficacy is the synergistic interaction between different components, which can enhance bioavailability, bioactivity, and overall health effects compared to isolated compounds. This study on rice and barley highlights how GABA, RS, and alkaloids interact to improve metabolic, neurological, and gastrointestinal health. Their synergistic effects are crucial in functional food development because some bioactive compounds improve the absorption of others; for example, resistant starch acts as a prebiotic, promoting gut microbiota and metabolizing GABA, potentially increasing its bioavailability. In addition, GABA (a neuroactive compound) and RS (a prebiotic) may work together to influence gut microbiota, which in turn affects neurotransmitter production and mental health. In barley, β-glucans and RS collectively improve insulin sensitivity and cholesterol metabolism, offering better outcomes than does either compound alone.

One of the key challenges in cereal genomics is bridging the genetic gap between rice (a model monocot with a well-annotated genome) and barley (a more complex, diploid Triticeae crop with a large genome). Comparative genomics allows researchers to identify conserved and divergent quantitative trait loci (QTLs) associated with critical agronomic traits. Among these, glutamate decarboxylase (*GAD*), starch synthase IIa (*SSIIa*), and cytochrome *P450* (*CYPs*) genes play crucial roles in stress response, grain quality, and metabolic pathways [27,28]. This in-depth analysis explores the genetic and functional differences between rice and barley by comparing QTLs and allelic variations in *GAD*, *SSIIa*, and *CYPs*, highlighting their implications for crop improvement. For rice genome structure and evolution, a small genome (~430 Mb), which is highly sequenced and annotated, belongs to the BEP clade (Bambusoideae, Ehrhartoideae, Pooideae) but diverged early from Triticeae [29]. Also, it has high gene density and synteny with other grasses, making it a model for cereal genomics. Meanwhile, the barley genome structure and evolution involves a large genome (~5.1 Gb), with ~80% repetitive elements, and is a member of the Triticeae tribe (along with wheat and rye) [30], which underwent recent polyploidization events, more complex regulatory mechanisms, and structural variations compared to rice.

Challenges in comparative genomics include synteny breakdown and the existence of colinear regions, extensive rearrangements, and transposable element expansions in barley reducing direct gene-to-gene comparisons. Certain gene families (e.g., CYPs) have undergone differential expansions in barley and rice. Divergent selection pressures for rice (tropical/subtropical) and barley (temperate/cold-adapted) have led to the evolution of distinct stress-responsive pathways. Rice *GADs* have been more extensively studied in terms of heat/drought responses, whereas barley *GADs* have been linked to cold tolerance. Barley may lack some regulatory elements found in rice, affecting GABA accumulation under stress. Starch Synthase *IIa* (*SSIIa*) catalyzes amylopectin elongation, affecting grain texture and cooking quality. Rice *SSIIa* has a stronger effect on cooking quality, while barley *SSIIa* is crucial for malting. Barley shows more allelic diversity, likely due to human selection for brewing traits. Cytochrome *P450* (*CYPs*) is involved in detoxification, hormone synthesis, and secondary metabolism. Rice *CYPs* are more studied in herbicide resistance, whereas barley *CYPs* are linked to Fusarium resistance (DON detoxification). Barley has expanded *CYP* clans (e.g., *CYP71*) for harsher temperate climate adaptations. The genetic gap between rice and barley is shaped by divergent evolution, genome complexity, and selection pressures. While rice serves as a genomic model, barley’s larger genome and adaptive traits present unique challenges and opportunities. Comparative analysis of *GAD*, *SSIIa*, and *CYP* QTLs reveals both conserved and divergent mechanisms underlying stress tolerance, grain quality, and metabolism. Bridging this gap through advanced genomics and biotechnology will enhance precision breeding for improved cereal crops. This comparative framework provides a foundation for future studies aiming to leverage rice genomic resources for barley improvement and vice versa.

In this review, we analyze the occurrence, biosynthesis, and functional significance of alkaloids in rice and barley in detail, emphasizing their potential as bioactive compounds significant to human health, plant defense, and agricultural applications. We examine the biochemical pathways, mutual interactions, and health implications of GABA, resistant starch, and alkaloids in rice and barley, emphasizing their roles in functional food development. By analyzing current research, we highlight the gaps in knowledge and future directions for harnessing these interactions to design tailored functional foods for enhancing metabolic, neurological, and gastrointestinal health.

## 2. Interaction of and Differences in Gene Loci Among Rice Functional Components

**Figure 1:**
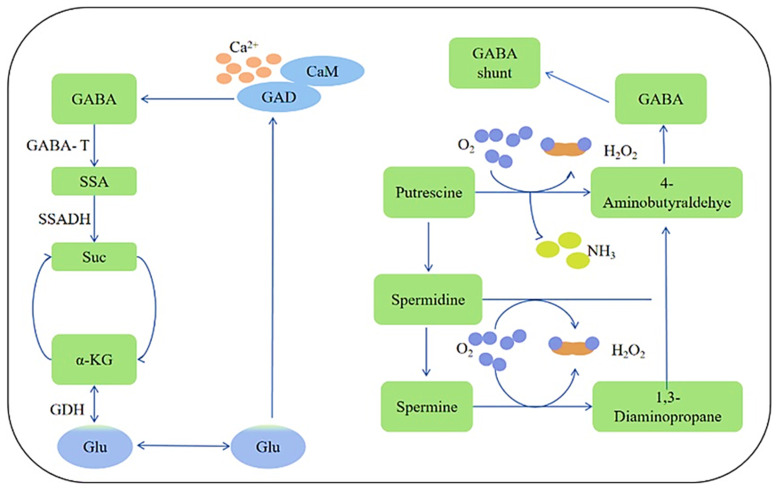
Metabolism process of GABA in plants.

Rice (*Oryza sativa*) contains several bioactive compounds; these components are governed by distinct genetic loci and metabolic pathways, and their interactions and differences in gene regulation contribute to rice’s functional properties [31,32]. GABA is a non-protein amino acid that functions as a major inhibitory neurotransmitter in the central nervous system of mammals [11]. It also plays significant roles in plants, including in stress responses, signaling, and growth regulation. GABA is found in various foods, including rice and barley, and its concentration can be influenced by processing methods, such as fermentation or germination. The compound can be measured according to [33], and Figure 1 explains its metabolism process in rice and barley [34].

**Figure 2:**
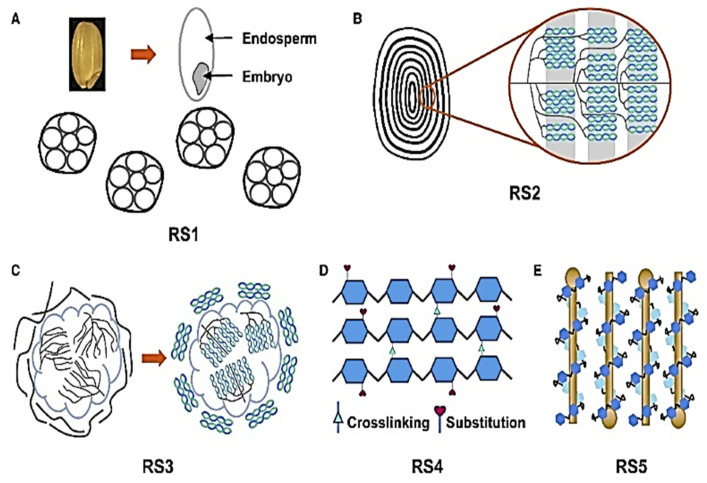
Types of resistant starch in plants. (**A**) RS1, a type of RS that is physically inaccessible to digestion. The starch granules are enclosed by cell walls or other tissues that prevent contact and reaction with amylase. (**B**) RS2, native starch granules with a compact crystalline structure that are relatively dehydrated. RS2 is tightly packed in a radial pattern in raw starch granules. (**C**) RS3, retrograded starches that are primarily formed via the gelatinization and retrogradation process during food processing and manufacturing. (**D**) RS4, chemically modified starch that generates new chemical bonds by substitution, esterification, or crosslinking. Through chemical modification, the molecular structure of starch is altered, thereby increasing its resistance to amylase. (**E**) RS5, a new type of RS that forms a complex of amylose and lipid. RS5 is usually generated in high-amylose starch cereals [37].

A kind of starch known as RS can function as a prebiotic in the colon and support gut health by avoiding digestion in the small intestine [35,36]. It is found in various foods, including rice and barley. Below is the detailed information about resistant starch in both rice and barley, and its types are shown in Figure 2 [37]. RS content can be measured according to the modified Goñi method [38].

Resistant starch (RS) is a fraction of starch that escapes digestion in the small intestine and functions similarly to dietary fiber, providing numerous health benefits. Among the five types of resistant starch, Type 5 (RS5) refers to the amylose–lipid complex (ALC), where amylose forms a helical inclusion complex with lipids, reducing enzymatic digestibility. RS5 is naturally present in cereals such as rice and barley and can be enhanced through processing techniques such as heat-moisture treatment (HMT) and annealing. The molecular basis of amylose–lipid complexes (ALCz) are amylose helix formation through amylose chains adopting a single helical V-type crystalline structure when complexed with endogenous lipids (e.g., free fatty acids, monoacylglycerols). Lipid binding through hydrophobic interactions between the lipid tail and the amylose helix core stabilizes the complex, reducing accessibility to amylolytic enzymes (α-amylase, amyloglucosidase). RS5 (amylose–lipid complex) in rice and barley represents a promising dietary component for improving metabolic health, gut microbiota balance, and glycemic control. While rice RS5 is highly influenced by processing, barley’s natural β-glucan content provides additional health synergies. Future research should focus on optimizing RS5 levels through agronomic practices, food processing, and genetic engineering to harness its full nutraceutical potential [39].

**Figure 3:**
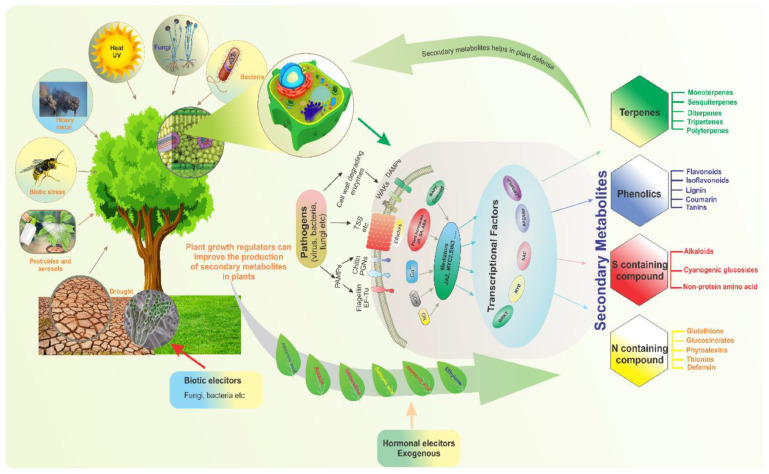
Plant growth and development are impacted by a variety of biotic and abiotic stressors [42].

Secondary metabolites called alkaloids are essential to plant physiology, defense systems, and interactions with the environment. Although they were traditionally studied in medicinal plants such as opium poppy and cinchona, alkaloids are increasingly being identified in cereal crops, including rice and barley [40,41]. These compounds contribute to the nutritional and functional properties of these grains, making them important for both human health and agricultural sustainability. This review explores the types of alkaloids found in rice and barley, their biosynthetic pathways, and their functional roles in plants and human diets (Figure 3) [42].

### 2.1. GABA in Rice

#### 2.1.1. Natural Occurrence

GABA is naturally present in rice, particularly in the germ and bran layers of brown rice. White rice contains significantly less of this compound compared to brown rice because the polishing process removes the germ and bran [43]. Brown rice has GABA contents ranging from 1 to 10 mg per 100 g [44], depending on the variety and germination conditions. In germinated brown rice (GBR), this content is significantly increased [45]. GBR can contain anything from 10 to 50 mg of GABA per 100 g or even more in some cases [46]. In white rice, GABA content is generally lower, typically less than 1 mg per 100 g, due to the removal of the bran layer during milling [47].

#### 2.1.2. Enhancement Through Germination

GABA is a non-protein amino acid and important for human health and plant stress responses. Soaking, germination, and fermentation are common methods to enhance the GABA content in rice, and fermentation using lactic acid bacteria or yeast can further increase the levels of this compound [48]. Its accumulation is influenced by environmental conditions, processing methods, and genetic factors [49]. Enhancing the GABA content in rice through germination, fermentation, or bio-fortification can improve its nutritional value, offering potential benefits such as anti-hypertensive, anti-diabetic, and neuroprotective effects [50,51]. Further research into GABA biosynthesis regulation and optimized processing techniques could lead to the development of functional rice varieties with enhanced health-promoting properties, contributing to both food security and human well-being. Germinated brown rice (GBR) or GABA rice is a popular functional food product. During germination, the activity of the enzyme glutamate decarboxylase (GAD) increases, converting glutamate into GABA. GABA levels in germinated brown rice can be 5–10 times higher than those in non-germinated brown rice [52]. Concentrations of GABA in different types of rice are shown in Table 1.

#### 2.1.3. GABA (γ-Aminobutyric Acid) Biosynthesis and Gene Loci

In rice (*Oryza sativa*), GABA (γ-aminobutyric acid) biosynthesis is prompted by L-glutamate through the action of the enzyme glutamate decarboxylase (GAD), which catalyzes the removal of carboxyl groups [58]. This reaction requires pyridoxal-5′-phosphate (PLP, a vitamin B6 derivative) as a cofactor [59,60,61]. GABA can be further metabolized in the GABA shunt (mitochondrial pathway) via GABA transaminase (GABA-T) catalysis, the conversion of GABA to succinic semialdehyde (SSA), succinic semialdehyde dehydrogenase (SSADH) catalysis, and the conversion of succinic semialdehyde (SSA) to succinate, thereby integrating into the tricarboxylic acid (TCA) cycle. GABA biosynthesis is regulated in rice through stress responses: the compound accumulates under hypoxia (flooding), heat stress, salinity, mechanical damage, and calcium signaling; Ca^2+^/calmodulin activates OsGAD1 under stress and developmental regulation; and GABA levels vary during seed germination and seedling growth.

##### Key Genes Involved in GABA Biosynthesis in Rice

GABA biosynthesis primarily occurs via the glutamate-decarboxylase-(GAD)-mediated decarboxylation of glutamate, followed by conversion via GABA transaminase (GABA-T). The key genes involved in this pathway include GAD family members (e.g., *OsGAD1*, *OsGAD2*), which regulate GABA production, and *OsGABA-T*, which modulates GABA catabolism. Additionally, the calcium/calmodulin (CaM)-binding domain in GAD enzymes links GABA synthesis to stress signaling. Several genes encode enzymes involved in GABA metabolism in rice (Table 2). The most critical ones include the following:

- Glutamate decarboxylase (GAD) genes: *OsGAD1* (LOC_Os03g15220) is found at chromosome number three; it converts glutamate to GABA and contains a calmodulin-binding domain (CaMBD), allowing calcium/calmodulin-mediated activation under stress (e.g., hypoxia, heat, salinity). *OsGAD2* (LOC_Os08g33690) is found at chromosome number eight; it functions similarly to OsGAD1 but may have different expression patterns.
- GABA transaminase (GABA-T) genes: *OsGABA-T1* (LOC_Os03g20310) is found at chromosome number three; it converts GABA to succinic semialdehyde (SSA). *OsGABA-T2* (LOC_Os01g48960) is found at chromosome number one; it functions as an alternative to GABA transaminase isoforms.
- Succinic semialdehyde dehydrogenase (SSADH) gene: *OsSSADH* (LOC_Os01g10940) is found at chromosome number one; it converts SSA to succinate, linking GABA metabolism to the TCA cycle.

The biosynthesis of GABA in rice is primarily mediated by *OsGAD1*/*2*, while GABA-T and SSADH regulate its catabolism. These genes are crucial for stress responses and nutritional enhancement in rice. The genetic manipulation of these loci can lead to high-GABA rice varieties with improved stress tolerance and health benefits.

##### Applications in Rice Breeding

Increasing GABA content in rice through breeding methods presents a promising approach to enhancing its nutritional value. This review explores various breeding strategies, such as conventional selection, marker-assisted breeding, and genomic approaches, in developing high-GABA rice varieties. Key genetic factors influencing GABA accumulation, including glutamate decarboxylase (GAD) activity and polyamine degradation pathways, are discussed. Additionally, the potential of mutation breeding and CRISPR-Cas9 gene editing to modulate GABA biosynthesis is highlighted. Successful case studies of biofortified rice cultivars with elevated GABA levels are examined, demonstrating the feasibility of breeding-based enhancement. Challenges, including yield trade-offs and regulatory considerations, are also addressed. In high-GABA rice varieties (e.g., germinated brown rice (GBR) used in functional foods), mutations in GABA-T or the overexpression of GAD can increase GABA content. Additionally, abiotic stress tolerance can enhance GABA synthesis and improve flood/drought resistance.

In plants, GABA accumulates in response to stress and plays a role in nitrogen metabolism, pH regulation, and defense mechanisms. Pigmented rice varieties, such as Venere rice (an Italian black rice), Thai jasmine black rice, and Chinese purple rice, have been found to contain significantly higher GABA levels compared to conventional white rice. This is attributed to their unique genetic makeup, anthocyanin-rich bran layers, and stress-induced metabolic pathways that enhance GABA biosynthesis. GABA is synthesized primarily via the glutamate decarboxylase (GAD) pathway, where the GAD decarboxylates L-glutamate into GABA. Pigmented rice varieties exhibit higher GAD enzyme activity due to genetic polymorphisms in GAD genes, enhanced stress responses (e.g., anaerobic germination, UV exposure, and cold storage), which upregulate GABA accumulation, and interaction with phenolic compounds, where anthocyanins (e.g., cyanidin-3-glucoside) may stabilize GABA or modulate its metabolic flux. Studies report GABA concentrations in pigmented rice ranging from 15–50 mg/100 g, compared to 2–10 mg/100 g in polished white rice. Germination (sprouting) and fermentation further amplify GABA levels, making germinated black rice a potent functional food.

#### 2.1.4. Health Benefits

GABA functions as a major inhibitory neurotransmitter in the central nervous system and has been linked to numerous health benefits. In rice (*Oryza sativa* L.), this compound accumulates through the decarboxylation of glutamate via glutamate decarboxylase (GAD), particularly under anaerobic conditions such as germination (sprouting) or fermentation. Recent studies highlight that GABA-rich rice, including germinated brown rice (GBR), exhibits enhanced nutritional and therapeutic properties. GABA has been associated with neuroprotective effects, including anxiety and stress reduction, improved sleep quality, and the potential alleviation of depression via the modulation of GABA receptors in the brain. Additionally, it demonstrates antihypertensive activity by inhibiting angiotensin-converting enzyme (ACE), thus aiding in blood pressure regulation. GABA-enriched rice also shows antidiabetic potential, improving insulin sensitivity and reducing oxidative stress in diabetic models. Furthermore, it enhances hepatic and renal function by mitigating oxidative damage and inflammation. GABA’s antioxidant properties contribute to reducing lipid peroxidation and enhancing cellular defense mechanisms, while its role in immune modulation may support metabolic and cardiovascular health. Fermented or germinated rice with elevated GABA levels also promotes gut health by influencing beneficial microbiota. Given these benefits, GABA-rich rice represents a functional food with promising applications in preventing and managing metabolic, neurological, and cardiovascular disorders [57,66]. Further research is needed to optimize GABA bioaccumulation in rice grains and validate its long-term health impacts in human clinical trials.

Human clinical benefits of GABA from pigmented rice include neuroprotective and antistress effects. Anxiolytic and sedative properties in dietary GABA from pigmented rice have been shown to reduce cortisol levels and improve sleep quality in clinical trials. Cognitive enhancement is achieved through GABA crossing the blood–brain barrier (albeit limitedly) and may enhance alpha-wave activity, improving relaxation and focus. Also, in terms of cardiovascular health, for blood pressure regulation, GABA acts as an ACE (angiotensin-converting enzyme) inhibitor, reducing hypertension in hypertensive patients. Antioxidant synergy through anthocyanins and GABA mitigates oxidative stress, lowering LDL oxidation and improving endothelial function. Pigmented rice varieties, particularly those with high anthocyanin content such as Venere rice, represent a natural reservoir of GABA, with demonstrated neuroprotective, cardiometabolic, and anti-inflammatory benefits. Further clinical research is needed to optimize bioavailability and establish dietary guidelines for maximal therapeutic effects. The integration of GABA-rich pigmented rice into functional diets could serve as a sustainable strategy for preventive healthcare.

#### 2.1.5. Differences in GABA Between Rice and Barley

Rice (*Oryza sativa*) and barley (*Hordeum vulgare*), despite being evolutionarily related, exhibit significant differences in GABA accumulation, regulation, and functional roles due to their distinct genetic backgrounds, environmental adaptations, and domestication histories. This in-depth comparative analysis explores the genetic, enzymatic, and metabolic mechanisms governing GABA biosynthesis, degradation, and physiological functions in rice and barley, highlighting key similarities, divergences, and potential biotechnological applications for crop improvement. The genetic and enzymatic basis of GABA metabolism for GABA is primarily synthesized via the glutamate decarboxylase (GAD) pathway, where GAD enzymes convert glutamate to GABA, followed by further metabolism through GABA transaminase (GABA-T) and succinic semialdehyde dehydrogenase (*SSADH*). While both rice and barley possess conserved GAD gene families, their structural and regulatory features differ. Rice contains five GAD genes (*OsGAD1-5*), with *OsGAD1* and *OsGAD2* being the most active in GABA synthesis under stress. These genes possess calmodulin-binding domains (CaMBDs), allowing calcium-mediated activation during abiotic stress. Meanwhile, barley has fewer GAD homologs (*HvGAD1-4*), with HvGAD1 showing strong induction under cold stress. Unlike rice, some barley GAD isoforms may lack CaMBD, suggesting alternative regulatory mechanisms. GABA levels in rice and barley are dynamically regulated under different environmental conditions. Rice GABA rapidly accumulates under heat, drought, and hypoxia, functioning as a signaling molecule to mitigate oxidative damage. High GABA rice varieties (e.g., Koshihikari) are prized for their nutritional benefits. On the other hand, barley GABA plays a critical role in cold acclimation and salinity tolerance, with elevated levels observed in response to low temperatures. Barley malting processes also induce GABA, influencing beer quality. GABA-rich rice (e.g., germinated brown rice) is promoted for its anti-hypertensive and neuroprotective effects. High-GABA barley is linked to improved malt fermentability and potential functional food applications. While rice and barley share core GABA metabolic pathways, their genetic regulation, stress adaptation strategies, and end-use qualities differ significantly. Understanding these distinctions provides a foundation for tailoring GABA-enriched cereals through precision breeding and genetic engineering, enhancing both agricultural resilience and nutritional value in a changing climate. Future research should focus on transgenic validation, multi-omics integration and field trials to optimize GABA traits in both crops. Later, we discuss GABA as a functional component in barley in detail.

### 2.2. Resistant Starch in Rice

#### 2.2.1. Types of Resistant Starch in Rice

Rice (*Oryza sativa*), a staple food for over half of the global population, contains varying levels of resistant starch depending on genetic, environmental, and processing factors. Resistant starch in rice is classified into five main types: RS1 (physically inaccessible starch) is found in whole or partially milled rice grains and trapped within intact cell walls, limiting enzymatic digestion; RS2 (resistant granules) is present in high-amylose rice varieties, where the native crystalline structure of starch (B-type polymorphs) resists digestion; RS3 (retrograded starch) is formed when cooked and cooled rice undergoes retrogradation, increasing amylose realignment and resistance to digestion; RS4 (chemically modified starch) is artificially produced through chemical treatments (e.g., cross-linking, esterification) to enhance resistance and is less common in natural rice; and RS5 (amylose–lipid complexes), often found in rice with higher lipid content, is produced by starch–lipid interactions that reduce digestibility [36,67]. The proportion of these RS types in rice depends on varietal differences (e.g., high-amylose vs. waxy rice), processing methods (e.g., parboiling, cooling), and cooking techniques. High-amylose rice cultivars, such as those with *ALK* gene mutations, tend to have higher RS2 and RS3 contents. Additionally, post-harvest treatments such as fermentation and hydrothermal processing can further modify RS levels. Understanding these variations is crucial for developing functional rice products with enhanced health benefits. This review explores the mechanisms, influencing factors, and potential applications of resistant starch in rice for improved human nutrition [68].

#### 2.2.2. Factors Affecting Resistant Starch Content

There are many factors affecting resistant starch content [69,70], including rice variety; some varieties, such as high-amylose rice (e.g., basmati or parboiled rice), have higher resistant starch contents compared to low-amylose rice (e.g., jasmine or sticky rice). Cooking rice and then cooling it increases resistant starch content due to retrogradation, through which starch molecules reorganize into a more resistant form [37,69,70]. Additionally, parboiled rice retains more resistant starch than does regular milled rice. Concentrations of resistant starch in different types of rice are shown in Table 3. There are some strategies to enhance RS, including genetic engineering (the knockout of *SBEIIb* or overexpression of *GBSSI* increases RS [37,71]) and mutagenesis (high-amylose mutants (e.g., “ae” mutants) contain elevated RS) [72,73]. Processing methods, including parboiling, cooling, and fermentation, can induce RS formation (RS3) [74,75]. Resistant starch in rice is primarily controlled by genes involved in amylose synthesis (*Wx*), starch branching (*SBEIIb*) [76], and starch structure modification (*SSIIa*, *SSIIIa*) [72,77]. Identifying and manipulating these key loci through breeding or biotechnology can enhance RS content, improving the nutritional quality of rice. Future research should focus on fine-mapping QTLs and developing high-RS rice varieties for better dietary health benefits.

#### 2.2.3. Resistant Starch Biosynthesis and Gene Loci

Like dietary fiber, RS is a form of dietary starch that evades digestion in the small intestine and offers health advantages such as better glycemic management, altered gut flora, and a lower risk of metabolic diseases. Although rice (*Oryza sativa* L.), a staple food for more than half of the world’s population, normally has a low RS content, some genotypes and processing techniques can increase it. Resistant starch formation in rice depends on starch structure, which is determined by the interplay of multiple biosynthetic enzymes [70,82]. The key steps in RS biosynthesis include the following: increasing amylose content, as high-amylose starch is more resistant to digestion due to its linear structure and reduced enzymatic accessibility; starch branching, as reduced branching (controlled by starch branching enzymes, SBEs) leads to longer amylose chains, increasing RS; starch retrogradation, as upon cooling, the realignment of starch molecules (particularly amylose) forms resistant crystalline structures (RS3); and phosphorylation, as phosphate esters in starch (mediated by Glucan Water Dikinase, GWD) can influence RS formation.

##### Key Genes and Loci Controlling RS

Several QTLs and genes have been identified that influence RS content in rice [83,84].

A. Major genes involved in RS biosynthesis

The biosynthesis of RS is a complex process governed by multiple enzymes and regulatory genes involved in starch metabolism. Key genes, such as *GBSSI* (granule-bound starch synthase I), encoded by the Waxy (*Wx*) gene, determine amylose content, a major determinant of RS formation. Mutations in *Wx* lead to varying amylose levels, influencing RS content. Additionally, *SSIIa* (starch synthase IIa) and *SBEIIb* (starch branching enzyme IIb) are critical in amylopectin chain elongation and branching, with their suppression resulting in higher RS accumulation. Other important genes include *ISA1* (Isoamylase 1) and *PUL* (Pullulanase), which regulate starch debranching, and their downregulation increases RS by reducing starch digestibility. Furthermore, transcription factors such as *RSR1* (Rice Starch Regulator 1) modulate the expression of starch biosynthetic genes, indirectly affecting RS content. Recent advances in genome editing (e.g., CRISPR-Cas9) have enabled precise modifications in these genes to enhance RS levels in rice cultivars. Understanding the genetic and molecular mechanisms underlying RS biosynthesis provides valuable insights for developing high-RS rice varieties with potential nutritional and therapeutic benefits. The biosynthesis pathway is discussed in detail below:

1. Genes in the *Waxy* (*Wx*) group (e.g., GBSSI, granule-bound starch synthase I) are found at chromosome number six, and these genes encode the enzyme responsible for amylose synthesis [85,86].
2. Starch branching enzymes (SBEs) named *SBEI*, found at chromosome number five, and *SBEIIb*, found at chromosome number two, influence amylopectin branching, in addition to the knockout or suppression of *SBEIIb*, and they increase amylose and RS (e.g., sbe3-rs mutant) [87,88].
3. Pullulanase (*ISA1*, Isoamylase 1) is found at chromosome number eight and affects starch debranching; mutations can alter starch structure and RS content [89].
4. *SSIIa* (starch synthase IIa) is found at chromosome number six and potentially influences amylopectin chain length; its variants affect RS formation [77,90].
5. *SSIIIa* (starch synthase IIIa) is found at chromosome number eight and can modify starch granule morphology; its mutations can increase RS [90,91].

B. Key QTLs associated with RS

Understanding the genetic basis of RS content is crucial for developing high-RS rice varieties through molecular breeding. Key QTLs associated with RS in rice can be identified by integrating genomic approaches and biochemical characterization. Recent advances in QTL mapping and genome-wide association studies (GWASs) have revealed several genomic regions influencing RS accumulation. Major QTLs have been identified at chromosomes 6, 8, and 11, harboring genes involved in starch biosynthesis, such as *GBSSI* (*Waxy*), *SSIIa*, and *SBEIIb*. Polymorphisms in these genes significantly impact amylose content and starch structure, directly affecting RS levels. Additionally, novel QTLs linked to starch degradation and retrogradation processes have been discovered, highlighting the complex genetic regulation of RS. This review consolidates findings from multiple studies, emphasizing the role of QTLs in RS variation across diverse rice germplasms. By elucidating the genetic architecture of RS, this research provides a foundation for developing functional rice varieties with improved nutritional benefits. Several QTLs have been mapped in rice populations [92,93,94], including qRS7 (found at chromosome seven), associated with high RS in indica rice, and qRS6 (found at chromosome six), which co-localizes with *Wx* and *SSIIa*. Additionally, qAC2 (found at chromosome two) is linked to amylose content and RS.

#### 2.2.4. Health Benefits

RS is a type of dietary starch that escapes digestion in the small intestine and ferments in the colon, functioning similarly to dietary fiber. It has gained significant attention due to its potential health benefits, including improved glycemic control, enhanced gut microbiota composition, and a reduced risk of chronic diseases such as obesity, type 2 diabetes, and colorectal cancer. Rice (*Oryza sativa*) is a major source of carbohydrates but typically contains low levels of RS. However, processing methods such as cooking and cooling, parboiling, and high-amylose rice breeding can increase RS content. This review explores the mechanisms by which RS exerts its health benefits, focusing on its role in promoting the production of short-chain fatty acids (SCFAs), particularly butyrate, which supports colonocyte health, reduces inflammation, and enhances insulin sensitivity. Additionally, RS modulates the gut microbiome by favoring beneficial bacteria such as Bifidobacterium and Lactobacillus while inhibiting pathogenic species. In rice, RS content can be enhanced through genetic modification, enzymatic treatments, and optimized cooking techniques, offering a practical approach to improving its nutritional value. The potential of RS-rich rice as a functional food is discussed, emphasizing its ability to lower postprandial glucose levels, improve satiety, and contribute to metabolic health. Future research should focus on optimizing RS levels in rice varieties and evaluating long-term health impacts in diverse populations. Enhancing RS in rice presents a promising strategy to addressing global health challenges related to metabolic disorders and gut health while accommodating cultural dietary preferences [14,95,96].

#### 2.2.5. Resistant Starch in Rice and Barley

Rice (*Oryza sativa*) and barley (*Hordeum vulgare*) are two major cereal crops with distinct RS profiles due to differences in their genetic, biochemical, and structural properties. While rice is predominantly a low-RS staple, barley contains higher RS content, particularly in its waxy and high-amylose varieties. This review provides an in-depth comparison of RS in rice and barley, a focus on the following: the genetic and molecular basis—key genes such as *GBSSI* (Granule-Bound Starch Synthase I), *SSIIa* (Starch Synthase IIa), and *SBEIIb* (Starch Branching Enzyme IIb) regulate amylose-to-amylopectin ratios, which directly influence RS formation, while rice mutants such as high-amylose rice (e.g., RS111) and barley genotypes (e.g., Himalaya 292) exhibit elevated RS due to altered starch biosynthesis pathways; starch structure and composition—barley starch contains more amylose (up to 40% in high-amylose types) and a higher proportion of resistant fractions (RS2 and RS3) compared to most rice varieties, and the presence of β-glucan in barley further enhances its RS properties, whereas rice starch is more readily digestible unless chemically or physically modified; environmental and processing effects—post-harvest processing (e.g., parboiling in rice, roasting in barley) and cooking methods significantly impact RS retention, and barley’s thicker cell walls and higher fiber content contribute to its superior RS stability under heat treatment compared to rice; health and nutritional implications—barley’s higher RS content is linked to stronger prebiotic effects, improved insulin sensitivity, and greater satiety than rice; yet, biofortified rice varieties (e.g., through CRISPR-edited Waxy alleles) are emerging as promising alternatives for increasing RS in populations dependent on rice as a staple. This comprehensive analysis highlights the potential of leveraging barley’s natural RS advantages while exploring genetic engineering and processing techniques to improve RS in rice. The findings provide a roadmap for developing next-generation high-RS cereals to address global nutritional challenges. Later we discuss in detail resistant starch as a functional component in barley.

### 2.3. Alkaloids in Rice

#### 2.3.1. Types of Alkaloids in Rice

Alkaloids are a diverse group of nitrogen-containing secondary metabolites found in various plants, including rice (*Oryza sativa* L.). While rice is primarily known as a staple food crop, it also contains several biologically active alkaloids that contribute to its pharmacological and nutritional properties. Rice contains several alkaloids, although at lower levels compared to those in medicinal plants [97,98,99]. The primary alkaloids identified in rice include gramine, a tryptophan-derived alkaloid with insecticidal and antifungal properties; hordenine, a phenethylamine alkaloid known for its antioxidant and antimicrobial activities; and indole alkaloids, derived from tryptophan, as well as isoquinoline and diterpenoid alkaloids, which are predominantly found in different parts of the plant, such as the husk, bran, and leaves. Indole alkaloids, such as tryptamine and its derivatives, play a role in plant defense mechanisms. Isoquinoline alkaloids, including nuciferine and liensinine, are primarily found in rice husks. Additionally, diterpenoid alkaloids, such as oryzalides, are unique to rice and contribute to its resistance against pathogens. The presence of these alkaloids in rice not only influences its medicinal value but also impacts food safety, as some may exhibit toxicity at high concentrations. Understanding the types, distribution, and biological functions of alkaloids in rice is crucial for optimizing its health benefits while minimizing potential risks.

#### 2.3.2. Biosynthesis of Alkaloids in Rice

The biosynthesis of alkaloids in rice (*Oryza sativa*) is governed by intricate metabolic pathways that are frequently upregulated in response to various stress conditions. In rice, the production of alkaloids involves multi-step enzymatic processes that originate from primary metabolic precursors, such as amino acids (e.g., tryptophan, tyrosine, and lysine) [100,101], and are regulated by a network of genes encoding key biosynthetic enzymes, including cytochrome P450 monooxygenases, methyltransferases, and oxidoreductases. Recent advances in genomics and metabolomics have unveiled stress-inducible transcription factors and signaling molecules, such as jasmonic acid and salicylic acid, which modulate alkaloid biosynthesis pathways. Furthermore, ecological and evolutionary perspectives suggest that the diversification of alkaloid structures in rice may enhance adaptive fitness under fluctuating environmental pressures [102]. Understanding the regulatory mechanisms and functional roles of alkaloid biosynthesis in rice not only provides insights into plant stress responses but also opens avenues for developing stress-resistant crop varieties through metabolic engineering. This review synthesizes current knowledge on the biochemical pathways, genetic regulation, and ecological significance of alkaloid production in rice, highlighting its potential applications in sustainable agriculture. The biosynthesis of alkaloids in rice involves complex pathways that are often induced under stress conditions [42,103,104,105]. The key steps include decarboxylation and methylation, enzymatic modifications that convert precursors into bioactive alkaloids, and regulation via environmental stress, such as pathogen attack, herbivory, and abiotic stress, which can upregulate alkaloid biosynthesis.

##### Major Classes of Alkaloids and Their Biosynthetic Pathways

A. Terpenoid indole alkaloids (TIAs; derived from tryptophan). The tryptophan pathway is a precursor for many alkaloids, including gramine and indole alkaloids (e.g., vinblastine, vincristine, strychnine) [106,107]. Key enzymes are tryptophan decarboxylase (TDC), which converts tryptophan to tryptamine; strictosidine synthase (STR), which condenses tryptamine with secologanin; and cytochrome P450s (e.g., CYP72A1), which modify strictosidine into complex TIAs.
B. Benzylisoquinoline alkaloids (BIAs; derived from tyrosine), e.g., morphine, codeine, and berberine [108,109,110]. Key enzymes are tyrosine decarboxylase (TYDC), which converts tyrosine to dopamine; norcoclaurine synthase (NCS), which condenses dopamine with 4-HPAA; berberine bridge enzyme (BBE), which forms (S)-reticuline; and CYP719B1 (canadine synthase), which produces berberine precursors.
C. Tropane alkaloids (derived from ornithine/arginine), e.g., atropine, scopolamine, and cocaine [111]. Key enzymes are ornithine decarboxylase (ODC), which produces putrescine; putrescine N-methyltransferase (PMT), which methylates putrescine; and hyoscyamine 6β-hydroxylase (H6H), which converts hyoscyamine to scopolamine.
D. Purine alkaloids (derived from xanthosine), e.g., caffeine and theobromine [112,113]. Key enzymes are xanthosine methyltransferase (XMT), which methylates xanthosine, and caffeine synthase (CS), which converts theobromine to caffeine.

#### 2.3.3. Biotechnological Application

Alkaloids have garnered significant attention in biotechnology due to their pharmacological and agrochemical properties. In rice (*Oryza sativa* L.), alkaloids play crucial roles in defense mechanisms against pathogens, pests, and environmental stressors, making them promising candidates for biotechnological applications. Recent advances in metabolic engineering and synthetic biology have enabled the manipulation of alkaloid biosynthetic pathways to enhance rice resilience, nutritional quality, and medicinal value. This review explores the biotechnological potential of alkaloids in rice, focusing on their biosynthesis, genetic regulation, and metabolic engineering strategies. Additionally, CRISPR-Cas9 and RNA interference (RNAi) technologies have been employed to modulate endogenous alkaloid production, improving stress tolerance, and reducing dependency on chemical pesticides. Furthermore, alkaloid-enriched rice varieties hold promise for nutraceutical applications, offering bioactive compounds with antioxidant, anti-inflammatory, and anticancer properties. Challenges such as yield penalties, metabolic trade-offs, and regulatory hurdles are also discussed. By integrating multi-omics approaches and precision breeding, the development of alkaloid-biofortified rice could revolutionize sustainable agriculture and functional food industries. Alkaloid biosynthesis is governed by multi-enzyme pathways encoded by specific gene loci, with key enzymes including STR, NCS, PMT, and XMT playing pivotal roles [114]. Understanding these genetic and biochemical mechanisms enables metabolic engineering for enhanced alkaloid production, benefiting pharmaceuticals and agriculture [115,116]. Future research should focus on pathway elucidation in non-model species and synthetic biology applications.

#### 2.3.4. Functional Roles of Alkaloids in Rice

Alkaloids play crucial functional roles in plant defense mechanisms, particularly in rice (*Oryza sativa* L.). As a staple crop, rice faces persistent biotic stressors, including herbivores, pathogens, and competing plants, which threaten yield and quality. Alkaloids contribute to rice defense through direct toxicity, antifeedant effects, and signaling modulation, deterring herbivores and inhibiting microbial proliferation. Notable rice alkaloids, such as gramine, hordenine, and avenanthramides, exhibit broad-spectrum antimicrobial and insecticidal properties, disrupting cellular processes in pests and pathogens. Additionally, alkaloids function as inducible defenses, with their biosynthesis often upregulated in response to herbivory or pathogen attack via jasmonic acid (JA) and salicylic acid (SA) signaling pathways. Recent studies highlight their role in allelopathy [117,118], suppressing competing weeds through root exudation. However, the metabolic cost of alkaloid production may produce a trade-off with growth, necessitating precise regulatory mechanisms. Advances in metabolomics and genetic engineering have unveiled key biosynthetic genes (e.g., cytochrome P450s and methyltransferases), enabling the development of alkaloid-enhanced rice varieties for sustainable pest management [110,119].

These bioactive compounds contribute to plant defense against biotic stressors, such as herbivores [120], pathogens [121], and competing plants, through their antimicrobial, antifeedant, and allelopathic properties. Additionally, alkaloids participate in abiotic stress responses, including those to drought [122], salinity, and heavy metal toxicity, by modulating oxidative stress through antioxidant activity and regulating stress-signaling pathways. Certain rice-associated alkaloids, such as gramine and hordenine, have been implicated in enhancing resistance to insect herbivory and microbial infections. Furthermore, alkaloids may interact with phytohormones, such as jasmonic acid and salicylic acid, to fine-tune defense mechanisms. Recent advances in metabolomics and genetic engineering have uncovered key biosynthetic pathways responsible for alkaloid production, offering potential for developing stress-resilient rice varieties [123].

Alkaloids play crucial functional roles in rice (*Oryza sativa*), particularly in relation to human health. While primarily known for their defensive properties against herbivores and pathogens in plants, certain rice-derived alkaloids exhibit significant bioactive potential, influencing human physiology in both beneficial and detrimental ways. Positive aspects include some rice alkaloids’ possession of antioxidant, anti-inflammatory, and anticancer properties, contributing to disease prevention and health promotion. For instance, compounds such as tricin and gramine have demonstrated chemo-preventive effects by modulating cellular signaling pathways linked to oxidative stress and carcinogenesis. Conversely, certain alkaloids may act as antinutritional factors, interfering with nutrient absorption or exhibiting mild toxicity at high concentrations. Additionally, the presence of alkaloids in rice can influence metabolic health, with some studies suggesting their role in glucose regulation and neuroprotection. However, the mechanisms underlying these effects remain understudied, necessitating further research to elucidate their pharmacokinetics, optimal bioactive concentrations, and potential interactions with other dietary components. Understanding the dual nature of rice alkaloids both as health-promoting agents and as presenting possible risks can guide dietary recommendations, biofortification strategies, and pharmacological applications, ultimately enhancing the nutritional and medicinal value of rice as a global staple food.

#### 2.3.5. Comparisons of Alkaloids Between Rice and Barley

While alkaloid diversity has been extensively studied in dicots, its presence and biosynthetic pathways in monocots, particularly cereals such as rice (*Oryza sativa*) and barley (*Hordeum vulgare*), remains less characterized. This review provides an in-depth comparative analysis of the alkaloid biosynthesis, diversity, and functional significance in rice and barley, highlighting key genetic, enzymatic, and metabolic differences between these two agriculturally important crops. Rice and barley exhibit distinct alkaloid profiles due to divergent evolutionary pressures and ecological adaptations. Barley, a temperate crop, produces gramine and hordenine—two major alkaloids with demonstrated roles in herbivore deterrence and fungal resistance. These compounds are derived from tryptophan and tyrosine, respectively, via specialized cytochrome *P450* (*CYP*) and methyltransferase enzymes. In contrast, rice primarily accumulates benzoxazinoid-related compounds and simple indole alkaloids, which contribute to pest resistance and allelopathy. The presence of benzoxazinoids in certain wild rice species, but their absence in cultivated rice suggests domestication-related loss, whereas barley has retained its alkaloid biosynthesis pathways due to strong selection for stress resilience. Genomic comparisons reveal that key alkaloid biosynthetic genes, particularly those encoding CYPs, decarboxylases, and O-methyltransferases, have undergone lineage-specific expansions in barley. For instance, the tryptamine N-methyltransferase (*HvTMT*) gene cluster is unique to barley and underlies gramine biosynthesis. Rice, however, lacks this pathway but possesses alternative detoxification-related *CYPs* (e.g., *CYP79A1*) that contribute to indole alkaloid diversification. Additionally, comparative transcriptomic studies indicate that barley alkaloid synthesis is highly inducible under biotic stress, whereas rice relies more on flavonoid and terpenoid defenses. The regulatory networks controlling alkaloid production also differ significantly. Barley employs jasmonate (JA)- and abscisic acid (ABA)-mediated signaling to upregulate alkaloid biosynthesis, whereas rice uses a broader array of phytohormones, including salicylic acid (SA), to modulate secondary metabolism. Furthermore, recent advances in metabolomics and genome-wide association studies (GWAS) have identified quantitative trait loci (QTLs) linked to alkaloid variation in barley (e.g., Alk1.1 on chromosome 2H), while analogous studies in rice remain limited. This comparative analysis underscores the evolutionary trade-offs between domestication and defense in cereals. Barley’s retention of alkaloid pathways highlights its ecological adaptation to temperate climates with higher pest pressures, whereas rice’s shift toward alternative defense mechanisms reflects its subtropical origins and human selection for palatability. Future research should explore metabolic engineering strategies to transfer beneficial alkaloid traits between these crops, potentially enhancing stress resilience without compromising agronomic performance. Understanding these differences provides a foundation for improving cereal crop defenses through targeted breeding and biotechnological approaches. Later we discuss alkaloids as a functional component in barley in detail.

### 2.4. Interactions Among GABA, RS, and Alkaloid Pathways

The interplay between GABA, resistant starch, and alkaloid biosynthesis pathways represents a complex and underexplored nexus in plant metabolism and human health. GABA, a key inhibitory neurotransmitter in mammals, also functions as a signaling molecule in plants, modulating stress responses and carbon–nitrogen balance. Resistant starch, a fermentable dietary fiber, influences gut microbiota composition and short-chain fatty acid (SCFA) production, indirectly affecting systemic GABAergic activity through the gut–brain axis. Meanwhile, alkaloids—nitrogen-containing secondary metabolites—often share biosynthetic precursors with GABA, particularly via glutamate and ornithine-derived pathways. Emerging evidence suggests that resistant starch fermentation may alter the availability of this compound by modulating microbial GABA metabolism or host glutamate decarboxylase (GAD) activity. Conversely, certain alkaloids can interact with GABA receptors, potentially competing with endogenous GABA or influencing its synthesis. This tripartite interaction has implications for metabolic health, neuroprotection, and stress adaptation in both plants and animals. For instance, GABA-enriched foods (e.g., fermented products or germinated grains) may synergize with resistant starch to enhance gut–brain signaling, while alkaloids from medicinal plants could either potentiate or inhibit GABAergic effects. This review synthesizes the current knowledge on the molecular crosstalk between these pathways, highlighting gaps in understanding how dietary components (resistant starch) and phytochemicals (alkaloids) collectively modulate GABA homeostasis.

The genetic loci controlling GABA, RS, and alkaloids in rice are distinct but may interact through shared precursors and stress responses [62,124]. Understanding these differences helps in breeding rice varieties with enhanced functional properties, such as high-GABA rice for relaxation, high-RS rice for diabetes management, and alkaloid-rich rice for medicinal applications (Table 4). Future research could explore multi-trait gene editing to develop rice with optimized levels of all three components.

## 3. Interaction of and Differences in Gene Loci Among Barley Functional Components

Barley (*Hordeum vulgare*) contains several bioactive compounds, including GABA, RS, and alkaloids, which contribute to its nutritional and functional properties [128]. These components are regulated by distinct genetic loci and metabolic pathways, yet their interactions and differences in gene regulation remain underexplored [7,129,130]. In this review, we examines the key gene loci associated with GABA biosynthesis (e.g., *GAD* genes), RS formation (e.g., *SSIIa*, *SBEIIb*), and alkaloid production (e.g., *TYDC*, *CYP450s*), highlighting their chromosomal locations (e.g., GABA QTLs on chromosome 2H, RS-related genes on 7H) and metabolic crosstalk. We discuss how environmental stressors influence these pathways and explore potential pleiotropic effects through which mutations in one pathway may impact another. Understanding these genetic mechanisms can guide the breeding of barley varieties with enhanced functional benefits, such as stress-resistant high-GABA barley, low-glycemic-index high-RS barley, and bioactive alkaloid-rich barley for nutraceutical applications. This synthesis provides a foundation for future research on multi-trait genetic improvement in barley.

### 3.1. GABA in Barley

#### 3.1.1. Natural Occurrence

Barley contains GABA, primarily in the outer layers of the grain. Similarly to that in rice, the concentration of GABA in barley can be increased through specific processing techniques [131]. GABA in barley grain is generally less abundant than that in rice, typically ranging from 0.5 to 5 mg per 100 g [132,133]. Nonetheless, germination can increase the GABA content in barley as well as that in rice. Germinated barley can have GABA levels ranging from 5 to 20 mg per 100 g, depending on the germination conditions [134,135].

#### 3.1.2. Enhancement Through Germination

Like that in germinated rice, GABA content in germinated barley can significantly increase by several times due to the activation of GAD during the germination process [136]; concentrations of this compound in different types of barley are shown in Table 5 [57,133,136]. Germination and fermentation are effective methods to boosting GABA content in barley. Barley melt, produced via controlled germination, is a common source of GABA in brewing and food industries. During barley germination (malting), GABA levels increase due to the activation of HvGAD1/HvGAD2 in the aleurone layer [137] and the breakdown of glutamine and glutamate reserves. GABA-rich malt is beneficial in brewing and functional foods. There are some conditions that GABA biosynthesis relies on: GAD is highly active at a low pH (e.g., during hypoxia in roots); calcium signaling (Ca^2+^/CaM) binding enhances GAD activity under stress; and transcriptional control GABA shunt genes are upregulated by abiotic stress (e.g., drought, salinity, cold), germination (with high GABA levels in malt), and pathogen attack (through defense signaling) [138,139].

#### 3.1.3. GABA (γ-Aminobutyric Acid) Biosynthesis and Gene Loci

GABA as a signaling molecule in plants, plays roles in stress responses (e.g., those to drought, salinity, and hypoxia), carbon–nitrogen balance, and defense mechanisms [64,142]. In barley (*Hordeum vulgare*), GABA accumulates under various abiotic stresses and during germination (malting). The GABA shunt, which circumvents the tricarboxylic acid cycle, is the main process used to generate GABA [51,64]. The key steps include glutamate decarboxylation, GABA conversion to succinate semialdehyde, and oxidation to succinate. Glutamate decarboxylase (GAD, EC 4.1.1.15) is the enzyme that oversees the reaction of L-Glutamate into GABA + CO_2_. With the aid of the cofactor Pyridoxal 5′-phosphate (PLP, vitamin B6-dependent), GAD is regulated by acidic pH (such as under hypoxia) and calcium/calmodulin (Ca^2+^/CaM) binding. In the reaction of GABA + α-ketoglutarate into succinate semialdehyde + L-Glutamate, the enzyme that produces GABA transaminase (GABA-T, EC 2.6.1.19) can substitute α-ketoglutarate with pyruvate or glyoxylate at the alternative substrate. For the oxidation to succinate, when succinate semialdehyde + NAD^+^ is reacted into succinate + NADH + H^+^, the enzyme that produces succinate semialdehyde dehydrogenase (SSADH, EC 1.2.1.24) re-enters the TCA cycle [143,144,145].

##### Key Genes Involved in GABA Biosynthesis in Barley

In barley (*Hordeum vulgare* L.), GABA accumulation is primarily regulated by the GABA shunt pathway, which involves key biosynthetic and catabolic enzymes. The biosynthesis of GABA is mainly catalyzed by glutamate decarboxylase (GAD), which converts L-glutamate into GABA in a calcium-/calmodulin-dependent manner. Barley possesses multiple *GAD* genes (*GAD1*, *GAD2*), with *GAD1* being particularly responsive to abiotic stresses such as hypoxia, salinity, and low pH. Another critical enzyme, GABA transaminase (GABA-T), and succinic semialdehyde dehydrogenase (SSADH) further modulate GABA levels by degrading GABA into succinate, integrating it into the TCA cycle. Recent studies have identified genetic variations in these key genes that influence GABA accumulation, offering potential targets for breeding barley with enhanced stress tolerance and nutritional benefits. Additionally, polyamine degradation pathways, involving enzymes such as diamine oxidase (DAO) and aminoaldehyde dehydrogenase (AMADH), contribute to alternative GABA biosynthesis under stress conditions. This review synthesizes current knowledge on the molecular genetics of GABA biosynthesis in barley, emphasizing the regulatory roles of *GAD*, *GABA-T*, and *SSADH* genes, and explores biotechnological approaches to manipulating GABA content for improved crop resilience and functional food applications. Several genes encode the enzymes of the GABA shunt in barley. These have been identified through genomic and transcriptomic studies, as shown in Table 6. GABA biosynthesis in barley is primarily mediated by the *HvGAD*, *HvGABA-T*, and *HvSSADH* genes, with regulation tied to stress responses and germination [137]. Understanding these genes can aid in developing stress-resilient barley varieties and improving malt quality.

#### 3.1.4. Health Benefits

Recent research has highlighted the potential health benefits of GABA-enriched foods, including stress reduction, improved sleep quality, antihypertensive effects, and neuroprotection. Barley (*Hordeum vulgare* L.), a widely cultivated cereal grain, has emerged as a promising candidate for GABA biosynthesis due to the high enzymatic activity of its glutamate decarboxylase (GAD), which converts glutamate into GABA under anaerobic conditions, such as during germination or fermentation. Studies indicate that GABA-rich barley products, such as germinated barley and fermented barley extracts, exhibit significant bioactive properties, including antioxidant, anti-inflammatory, and cardioprotective effects. Furthermore, GABA biosynthesis in barley has been linked to improved gut health via its modulation of the gut–brain axis and promotion of beneficial microbiota. The potential applications of GABA-enriched barley in functional foods and nutraceuticals are vast, offering a natural approach to mitigating chronic diseases such as hypertension, anxiety disorders, and metabolic syndrome. Additionally, GABA-rich barley is linked to improved cardiovascular health, stress reduction, and better sleep. It is also used in functional foods and beverages due to its potential to lower blood pressure and improve overall well-being [136,149].

#### 3.1.5. Comparison of GABA in Rice and Barley

Rice (*Oryza sativa*) and barley (*Hordeum vulgare*) are two major cereal crops that accumulate GABA under different physiological conditions, yet their GABA biosynthesis, regulation, and accumulation patterns exhibit both similarities and differences. This comparative review explores the GABA metabolic pathways in rice and barley, focusing on the key enzymes glutamate decarboxylase (GAD) and GABA transaminase (GABA-T), as well as factors influencing GABA levels, such as germination, fermentation, and environmental stresses. Rice, particularly brown and germinated rice, is known for its high GABA content, which can be further enhanced through anaerobic treatment or microbial fermentation. Barley, on the other hand, significantly accumulates GABA during malting and sprouting, making it valuable for brewing and functional food applications. While both crops rely on the GABA shunt for stress adaptation, barley exhibits a stronger GABA response to drought and salinity compared to rice, possibly due to differences in GAD isoforms and regulatory mechanisms. Additionally, genetic variations between rice and barley influence GABA-enriched product development, with rice being more commonly utilized in GABA-fortified foods. Both rice and barley can be enhanced to increase their levels of GABA through germination or fermentation. The exact concentration depends on the variety, processing methods, and conditions (e.g., temperature, pH, and treatment duration). GABA-rich rice and barley are used in functional foods, such as GABA rice tea, GABA barley tea, and fermented products. These products are marketed for their health benefits, particularly in Asian countries. Additionally, each grain provides additional nutrients, such as fiber, vitamins, and minerals, alongside GABA. Brown rice and whole barley are considered more nutritious due to their higher fiber and nutrient contents.

### 3.2. Resistant Starch in Barley

A form of dietary fiber called RS ferments in the colon after evading digestion in the small intestine. It offers several health advantages, such as better glycemic management, gut health, and a lower risk of colorectal cancer [150,151,152]. Barley (*Hordeum vulgare*) is a rich source of RS, particularly high-amylose varieties. This review summarizes the biochemical pathways of RS biosynthesis in barley, identifies key genes involved in RS formation, and discusses genetic and biotechnological approaches to enhance RS content. Understanding the genetic regulation of RS barley can facilitate the development of functional foods with enhanced nutritional value.

#### 3.2.1. Types of Resistant Starch in Barley

Barley (*Hordeum vulgare* L.) is a significant cereal grain known for its high content of dietary fiber, particularly RS, which confers various health benefits, including improved glycemic control, enhanced gut health, and a reduced risk of chronic diseases. Resistant starch, defined as the starch fraction that escapes digestion in the small intestine and ferments in the colon, is categorized into five main types (RS1–RS5), each with distinct structural and functional properties. In barley, resistant starch primarily exists as RS1 (starch that is physically inaccessible due to intact cell walls), RS2 (native granular starch with a high amylose content and a B- or C-type crystalline structure), and RS3 (retrograded starch formed after cooking and cooling). Additionally, RS4 (chemically modified starch) may be present in processed barley products, while RS5 (amylose–lipid complexes) is less common but can exist in barley due to interactions between starch and endogenous lipids. The proportion and bioavailability of these RS types in barley are influenced by genetic factors, environmental conditions, and processing methods. Recent advances in breeding and bioprocessing techniques aim to enhance RS content in barley cultivars, particularly RS2 and RS3, to maximize nutritional benefits. Understanding the distribution and behavior of different RS types in barley is crucial for developing functional foods with tailored health-promoting properties [153].

#### 3.2.2. Factors Affecting Resistant Starch Content

There are many factors affecting resistant starch content, including barley variety; hulled barley (with the outer bran intact) has a higher resistant starch content compared to pearled barley (polished to remove the bran). Additionally, less processed barley retains more resistant starch [154,155]. Concentrations of starch in different types of barley are shown in Table 7 [156,157,158,159]. Other factors influence resistant starch; its content in cooked and cooled barley ranges between 3 and 5%, whereas hulled barley has a higher resistant starch content compared to pearled barley [160]. Abundant nitrogen reduces RS, while phosphorus has a minimal effect, and retrogradation (RS3 formation) is enhanced by cooking and cooling.

#### 3.2.3. Resistant Starch Biosynthesis and Gene Loci

RS is classified into five types (RS1–RS5) based on its structure and resistance to digestion [161,162]. Barley naturally contains RS2 (granular starch in raw form) and RS3 (starch retrograded after cooking and cooling) [163]. High-RS varieties are nutritionally superior, making them valuable for diabetes management and digestive health [14,94,164]. Starch biosynthesis occurs in the amyloplast and involves four key enzymes [164,165,166,167]: ADP-glucose pyro-phosphorylase (AGPase) produces ADP-glucose, the substrate for starch synthesis; granule-bound starch synthase (GBSS) synthesizes amylose (linear α-1,4-glucan chains); starch branching enzyme (SBE) introduces α-1,6-branches to form amylopectin; and starch debranching enzyme (DBE) trims excessive branches for proper crystallization. High amylose content (a key determinant of RS) is influenced by reduced SBEII activity, which reduces branches and makes amylose more linear, and increased GBSS activity, which increases amylose content.

##### Key Gene Loci Controlling RS in Barley

Several genes regulate starch structure and RS content in barley, the most significant of which are shown in Table 8. Resistant starch in barley is primarily controlled by *HvGBSSI*, *HvSBEIIa*/*b*, and *HvISA1*, with high-amylose genotypes containing the greatest RS contents. Advances in gene editing and breeding are enabling the development of barley varieties with enhanced RS for improved human health. Future research should focus on optimizing agronomic practices and processing methods to maximize RS yield.

#### 3.2.4. Health Benefits

Barley, a nutrient-rich cereal grain, is a notable source of RS, particularly its whole and high-amylose varieties. This review explores the physiological effects of barley-derived resistant starch, emphasizing its role in improving metabolic health, gut microbiota composition, and disease prevention. Research indicates that barley RS enhances glycemic control by slowing glucose absorption, making it beneficial for individuals with insulin resistance or type 2 diabetes. Additionally, its fermentation in the colon produces short-chain fatty acids (SCFAs) such as butyrate, which supports colonocyte health, reduces inflammation, and may lower colorectal cancer risk. The prebiotic properties of barley RS promote the growth of beneficial gut bacteria (e.g., *Bifidobacterium* and *Lactobacillus*), improving gut barrier function and immune modulation. Furthermore, barley RS contributes to weight management by increasing satiety and reducing fat storage. Its ability to lower cholesterol levels through bile acid binding and SCFA production also highlights its cardiovascular benefits. Given these multifaceted advantages, incorporating barley-rich foods into diets could serve as a strategic approach to combating metabolic disorders, gastrointestinal diseases, and obesity [14,95,96].

### 3.3. Alkaloids in Barley

Alkaloids are nitrogen-containing secondary metabolites with diverse pharmacological and ecological roles, including defense against herbivores and pathogens [102,172]. While barley (*Hordeum vulgare*) is not a major alkaloid-producing crop compared to plants such as opium poppy or tobacco, it contains several bioactive alkaloids, such as gramine and hordenine, which contribute to its stress resilience and medicinal properties [173,174]. This review synthesizes current knowledge on alkaloid biosynthesis pathways in barley, identifies key gene loci involved in their production, and discusses genetic and biotechnological approaches to manipulating alkaloid content for improved crop resilience and nutraceutical applications.

#### 3.3.1. Types of Alkaloids in Barley

Barley, a widely cultivated cereal grain, is known for its rich contents of alkaloids [175,176,177], which are structurally diverse and perform significant biological activities. In barley, the most studied alkaloids include hordenine, known for its stimulant and antimicrobial properties. Gramine also contributes to barley’s defense mechanisms, and β-carboline alkaloids exhibit neuroprotective and antioxidant activities.

##### Alkaloids Biosynthesis and Gene Loci

- Alkaloids biosynthesis

The biosynthesis of alkaloids in barley has similarities with rice but has unique features [178,179].

- Gramine biosynthesis

Gramine, a bioactive indole alkaloid predominantly found in barley (*Hordeum vulgare*), exhibits significant defensive properties against herbivores and pathogens, contributing to plant stress resilience. Despite its ecological importance, the biosynthetic pathway and genetic regulation of gramine remain only partially elucidated [173,180]. Recent advances in genomics and metabolomics have identified key candidate genes involved in gramine biosynthesis, including putative tryptophan decarboxylases (TDCs) and methyltransferases (MTs), which likely catalyze the conversion of tryptophan to gramine via intermediate steps such as the formation of 3-aminomethylindole (AMI). Genetic mapping studies regarding barley have localized QTLs associated with gramine accumulation to chromosomes 2H, 5H, and 7H, implicating several cytochrome P450s (CYPs) and UDP-glycosyltransferases (UGTs) in its regulation. Furthermore, transcriptomic analyses under biotic stress conditions reveal the induction of specific biosynthetic genes, suggesting a jasmonate-mediated signaling pathway controlling gramine production. Comparative genomics with related Poaceae species highlights conserved enzymatic functions as well as species-specific gene duplications, which may explain differential gramine accumulation across cultivars [181,182].

- Hordenine biosynthesis

Hordenine (N,N-dimethyl-tyramine) is a bioactive alkaloid found in barley (*Hordeum vulgare*) and other related grasses, with potential pharmacological properties. Its biosynthesis involves the decarboxylation of tyrosine to tyramine via tyrosine decarboxylase (TyDC), followed by N-methylation via N-methyltransferases (NMTs), such as norcoclaurine-6-O-methyltransferase-like enzymes [183,184,185]. Recent advances in genomics and metabolomics have enabled the identification of candidate gene loci associated with hordenine biosynthesis in barley. Key QTLs have been mapped to specific chromosomal regions, particularly on chromosomes 2H and 5H, which harbor genes encoding enzymes involved in phenylpropanoid and alkaloid pathways. Transcriptomic analyses reveal the differential expression of these biosynthetic genes during barley germination, in which hordenine accumulation is highest. Furthermore, comparative genomics suggests the evolutionary conservation of hordenine-related genes in *Triticeae* species. Understanding the genetic regulation of hordenine biosynthesis could facilitate metabolic engineering for the enhanced production or the development of low-alkaloid barley varieties for improved food and feed safety [178,186,187].

- Avenanthramides (barley-specific phenolic alkaloids)

Avenanthramides (AVNs) are a unique class of phenolic alkaloids predominantly found in barley (*Hordeum vulgare*), known for their potent antioxidant, anti-inflammatory, and health-promoting properties. These compounds are synthesized via the phenylpropanoid pathway, via which hydroxycinnamoyl-CoA thioesters and anthranilic acid derivatives undergo condensation reactions catalyzed by hydroxycinnamoyl-CoA: hydroxy anthranilate N-hydroxycinnamoyl transferase (HHT) [188,189]. Recent genomic and transcriptomic studies have identified key gene loci associated with AVN biosynthesis in barley, including *HvHHT* and cytochrome P450 (*CYP*), genes involved in the modification of the AVN core structure. QTL mapping and genome-wide association studies (GWASs) have further revealed genetic variations influencing AVN accumulation, particularly on chromosomes 2H, 5H, and 7H. Additionally, the regulation of *AVN* biosynthesis is modulated by transcription factors such as those in the MYB and bHLH families, which respond to environmental stressors such as pathogen attack and UV radiation. Understanding the genetic and enzymatic mechanisms underlying AVN production provides valuable insights for biofortification strategies and the development of barley cultivars with enhanced nutraceutical value [190].

##### Key Gene Loci Controlling Alkaloids in Barley

Several genes regulate alkaloids in barley, the most significant of which are shown in Table 9. Barley produces several bioactive alkaloids, with gramine, hordenine, and avenanthramides being the most notable. Key genes (HvTDC, HvTyDC, HvHHT) have been partially characterized, but further research is needed to fully map their regulatory networks. Manipulating these pathways could enhance barley’s stress resilience and nutritional value, offering opportunities for sustainable agriculture and functional food development.

Barley (*Hordeum vulgare*), a key cereal crop, produces several bioactive alkaloids, including gramine, hordenine, and avenanthramides, which contribute to its resistance mechanisms and potential health benefits. Recent advances in genomics and metabolomics have identified key genes involved in alkaloid biosynthesis, enabling a deeper understanding of their regulation and applications in crop improvement as shown in Table 10. Gramine (indole alkaloid) functions as a defense compound toxic to insects and pathogens and biosynthesizes genes through tryptophan decarboxylase (TDC)—converting tryptophan to tryptamine and gramine synthase—catalyzing the methylation of tryptamine to form gramine. Hordenine (phenylethylamine alkaloid) has antimicrobial and allelopathic properties and biosynthesizes tyrosine decarboxylase (TYDC)—producing tyramine from tyrosine and N-methyltransferases (NMTs)—converting tyramine to hordenine. Avenanthramides (phenylalanine-derived alkaloids) functions as an antioxidant and anti-inflammatory compound and biosynthesizes genes through phenylalanine ammonia-lyase (PAL)—initiating the phenylpropanoid pathway and hydroxycinnamoyl-CoA transferases (HCTs)—synthesizing avenanthramide precursors. Alkaloid-related genes in barley govern critical defense and health-promoting compounds. Understanding their genetic regulation opens avenues for developing stress-resistant, nutritionally enhanced barley varieties with applications in sustainable agriculture and functional foods.

#### 3.3.2. Functional Roles of Alkaloids in Barley

Alkaloids provide defense mechanisms for barley, protecting it from pests and diseases, reducing the need for chemical pesticides. They also provide adaptation to abiotic stress by enhancing barley’s resilience to environmental challenges [179]. Alkaloids also confer nutritional and health benefits, contributing to the grain’s functional food properties, including antioxidant and anti-inflammatory effects [128].

#### 3.3.3. Comparative Analysis of Alkaloids in Rice and Barley

While both rice cereals and barley contain alkaloids, their profiles and concentrations differ significantly. In terms of diversity, barley contains more diverse alkaloids compared to rice. In terms of concentration, alkaloids are generally present at higher concentrations in barley, making it a more potent source of bioactive compounds. In terms of functional roles, both grains utilize alkaloids for defense and stress adaptation, but barley’s alkaloids are more extensively studied due to their pharmacological potential.

#### 3.3.4. Application in Human Health and Agriculture

- Human health

- Antioxidant properties: Alkaloids in rice and barley enhance the antioxidant capacity of these grains, potentially reducing oxidative stress in humans [202].
- Anti-inflammatory effects: Some alkaloids exhibit anti-inflammatory properties, which may benefit chronic disease prevention [203].
- Neuroprotective potential: β-carboline alkaloids in barley have shown promise in neuroprotection and mental health [204].

- Agriculture application

- Natural pesticides: Alkaloids can be harnessed for eco-friendly pest control in rice and barley cultivation [205].
- Stress-resilient crops: Understanding alkaloid biosynthesis can aid in developing stress-tolerant varieties of rice and barley [206].
- Functional foods: Enhancing alkaloid content through breeding or biotechnology can improve the nutritional value of these grains [207].

Rice and barley are not only staple foods but also rich sources of functional components that offer numerous health benefits. Incorporating whole-grain rice (e.g., brown rice, black rice) and barley into diets can provide essential nutrients, antioxidants, and bioactive compounds that support cardiovascular health, glycemic control, gut health, and overall well-being. Further research is needed to explore the full potential of these functional components and their applications in functional foods.

## 4. Conclusions

Rice (*Oryza sativa*) and barley (*Hordeum vulgare*) are two of the most important cereal crops globally, serving as staple foods for billions. Beyond their nutritional value, they are rich sources of bioactive compounds such as GABA, RS, and alkaloids, which contribute to their nutraceutical potential. A synergistic approach combining these compounds from both cereals could revolutionize functional food development, offering enhanced health benefits ranging from metabolic regulation to neuroprotection. GABA, a non-protein amino acid, acts as a major inhibitory neurotransmitter in the central nervous system, aiding stress reduction, blood pressure regulation, and improved sleep. In rice and barley, GABA accumulates under stress conditions (e.g., hypoxia, germination) via the glutamate decarboxylase (GAD) pathway. Key genes such as *OsGAD1*/*GAD2* (rice) and *HvGAD1* (barley) regulate GABA synthesis. Enhancing GABA content through targeted breeding or fermentation could amplify its anxiolytic and cardioprotective effects. Additionally, high-RS varieties of rice (e.g., high-amylose mutants such as *OsSSIIIa*) and barley (e.g., *HvGBSSI* mutants) have been developed by manipulating starch biosynthesis genes. Combining RS from both grains could enhance colonic fermentation, fostering a healthier gut microbiome. While alkaloids are less studied in cereals compared to medicinal plants, certain alkaloids in rice (e.g., tricin) and barley (e.g., gramine) exhibit antioxidant, anti-inflammatory, and anticancer properties. Biosynthetic pathways involve enzymes such as tryptophan decarboxylase (TDC) and cytochrome P450s, with genetic loci such as *OsTDC1* (rice) and *HvTDC* (barley) playing crucial roles. Optimizing alkaloid content could enhance the chemo-preventive potential of nutraceuticals derived from these grains. Future perspectives should focus on advancements in genomic editing (CRISPR-Cas9), fermentation biotechnology, and precision breeding, which can optimize the biosynthesis of these compounds. Identifying QTLs and regulatory genes will facilitate the development of highly nutraceutical rice and barley varieties. Furthermore, clinical trials validating synergistic effects will be essential for commercializing functional foods. In conclusion, integrating the nutraceutical properties of rice and barley presents a promising strategy for combating modern health challenges. By leveraging their complementary bioactive profiles, we can develop next-generation functional foods that promote holistic well-being, bridging the gap between agriculture and preventive medicine. Additionally, we discuss the health implications of these functional components, including their roles in reducing hypertension, managing diabetes, and exhibiting neuroprotective effects. Understanding the genetic differences between rice and barley in accumulating these compounds can guide biofortification strategies to enhance nutritional quality in cereal crops, ultimately benefiting human health and dietary outcomes. Our study has potentially wide implications, as we discuss the key functional components in both rice and barley and their potential health benefits. This review provides a detailed analysis of the types, structures, and functional roles of GABA, RS, and alkaloids in rice and barley, highlighting their nutritional significance, technological application, and potential for improving human health and agricultural sustainability. All the references were included in the manuscript related to our work, and some of them from our previous work at the department. We believe the results will be of interest to a wide readership including academics and researchers working in agriculture; genetics scientists, policy makers, and managers in government agencies; and agriculture and food security and policy makers.

## Figures and Tables

**Table 1:** GABA concentration in rice (mg/100 g dry weight).

Grain Type	Processing Method	GABA Concentration (mg/100 g)	Key Factors Influencing GABA Levels	Refs.
White Rice	Raw (unprocessed)	1.5–5.0	Milling makes levels of endogenous GABA low	[53,54,55,56,57]
Brown Rice	Raw (unprocessed)	5.0–15.0	Bran layer contains more GABA	
Germinated Brown Rice (GBR)	Soaking + germination (24–72 h)	25–350	Activation of glutamate decarboxylase (GAD)	
Fermented Rice	Lactic acid bacteria (LAB) fermentation	50–400	Microbial activity enhances GABA synthesis	
Black Rice	Germination	100–500	Anthocyanin-rich varieties contain more GABA	

**Table 2:** Summary of key GABA-related genes in rice.

Gene	Locus ID	Chromosome	Function	Refs.
OsGAD1	LOC_Os03g15220	3	Glutamate decarboxylase	[62,63,64,65]
OsGAD2	LOC_Os08g33690	8	Alternative GAD isoform	
OsGABA-T1	LOC_Os03g20310	3	GABA transaminase	
OsGABA-T2	LOC_Os01g48960	1	Alternative GABA-T isoform	
OsSSADH	LOC_Os01g10940	1	Succinic semialdehyde dehydrogenase	

**Table 3:** Resistant starch (RS) concentrations in rice (% of dry weight).

Grain Type	Processing Method	Resistant Starch Content (% DW)	Key Factors Influencing RS Levels	Refs.
White Rice (Cooked)	Normal cooking (boiled)	0.5–1.5%	High gelatinization reduces RS	[78,79,80,81]
Brown Rice (Cooked)	Normal cooking	1.0–2.5%	Higher fiber retains some RS	
Parboiled Rice	Pre-gelatinization and drying	2.0–4.0%	Retrogradation increases RS3	
Cooled Cooked Rice (Retrograded)	Cooking and refrigeration (4 °C, 12–24 h)	3.0–6.0%	Retrogradation forms RS3 (Type 3)	
High-Amylose Rice (e.g., RS Rice Varieties)	Raw preparation or cooking	5.0–15.0%	Genetically high amylose (25–30%) boosts RS2	

**Table 4:** Key differences among the functional components in rice.

Feature	GABA	Resistant Starch	Alkaloids	Refs.
Primary Function	Neurotransmission, stress response	Producing dietary fiber, glycin control	Defense, producing medicinal effects	[125,126,127]
Key Genes	GAD1, GAD2	SSlla, SBEllb	TYDc, CYP450	
Main Chromosome QTLs	1, 5, 8	6, 7	4, 11	
Inducing Factors	Hypoxia, germination	High amylose, enzymatic defects	Pathogen attack, stress	

**Table 5:** GABA concentration in barley (mg/100 g dry weight).

Grain Type	Processing Method	GABA Concentration (mg/100 g)	Key Factors Influencing GABA Levels	Refs.
Barley (Whole)	Raw preparation (no processing)	10–30	Levels are naturally higher than those in polished rice	[57,133,136,140,141]
Germinated Barley	Soaking + germination (48 h)	50–200	Optimal pH and temperature increase GABA	
Fermented Barley (Malt)	Controlled malting	80–300	Enzymatic activity during malting boosts GABA	

**Table 6:** Key genes involved in GABA biosynthesis.

Gene	Enzyme	Gene Locus (Barley Ref. Genome: Morex V3)	Characteristics	Refs.
HvGAD1	Glutamate decarboxylase (GAD)	HORVU.MOREX. r3.2HG0145020 (Chr. 2H)	Ca^2+^/CaM-binding domain, stress-responsive	[146,147,148]
HvGAD2	Glutamate decarboxylase (GAD)	HORVU.MOREX. r3.3HG0253870 (Chr. 3H)	Expressed in germinating seeds	
HvGABA-T1	GABA transaminase	HORVU.MOREX. r3.4HG0342100 (Chr. 4H)	Mitochondrial localization	
HvSSADH1	Succinate semialdehyde dehydrogenase	HORVU.MOREX. r3.5HG0456320 (Chr. 5H)	Converts SSA to succinate	

**Table 7:** Resistant starch (RS) concentrations in barley (% of dry weight).

Grain Type	Processing Method	Resistant Starch Content (% DW)	Key Factors Influencing RS Levels
Barley (Whole, Raw)	Unprocessed	3.0–8.0%	β-glucans and RS2 are at naturally high levels
Pearled Barley (Cooked)	Boiled	1.5–3.5%	Polishing reduces fiber and RS
Barley Flour (Baked)	Heat-treated (e.g., bread)	1.0–2.5%	Gelatinization lowers RS
Germinated Barley	Soaked/sprouted (48 h)	2.0–5.0%	Enzymes partially degrade RS
Barley Flakes (Rolled)	Steam-pressed and dried	4.0–7.0%	Processing preserves RS2

**Table 8:** Key gene loci controlling resistant starch in barley.

Gene	Enzyme	Function	Chromosomal Location (Morex V3)	Effect on RS	Refs.
HvGBSSI (Waxy)	Granule-bound starch synthase I	Amylose synthesis	HORVU.MOREX.r3.7HG0634210 (7H)	Increases RS	[168,169,170,171]
HvSBEIIa	Starch branching enzyme IIa	Amylopectin branching	HORVU.MOREX.r3.2HG0123400 (2H)	Increases RS	
HvSBEIIb	Starch branching enzyme IIb	Amylopectin branching	HORVU.MOREX.r3.2HG0123500 (2H)	Knocks out abundant amylose	
HvISA1 (DBE)	Isoamylase 1	Debranching enzyme	HORVU.MOREX.r3.4HG0387650 (4H)	Affects starch crystallinity	
HvAGP-L	ADP-glucose pyrophosphorylase (large subunit)	ADP-glucose synthesis	HORVU.MOREX.r3.1HG0056780 (1H)	Modulates starch yield	

**Table 9:** Key gene loci controlling alkaloids in barley.

Alkaloid	Key Gene	Enzyme	Chromosomal Location (Morex V3)	Function	Refs.
Gramine	HvTDC	Tryptophan decarboxylase	HORVU.MOREX.r3.5HG0452100 (5H)	Converts tryptophan → tryptamine	[178,191,192,193]
	HvNMT	N-methyltransferase	Unknown (predicted 2H or 7H)	Methylates tryptamine	
Hordenine	HvTyDC	Tyrosine decarboxylase	HORVU.MOREX.r3.3HG0278900 (3H)	Converts tyrosine → tyramine	
	HvBBE	Berberine bridge enzyme	HORVU.MOREX.r3.6HG0523400 (6H)	Oxidizes N-methyltyramine → hordenine	
Avenanthramides	HvHHT	Hydroxycinnamoyltransferase	HORVU.MOREX.r3.4HG0391200 (4H)	Forms avenanthramide conjugates	

**Table 10:** Alkaloid-Related Genes in Barley and Their Functions.

Gene Name	Gene ID/Locus	Alkaloid Pathway	Function	Impact/Phenotype	Refs.
HvNMT (*N-methyltransferase*)	MLOC_11101 (Chromosome 2H)	Gramine biosynthesis	Catalyzes the methylation of tryptamine to form *N*-methyltryptamine (precursor to gramine)	High expression in leaves; contributes to aphid resistance	[194,195]
HvTDC (*Tryptophan decarboxylase*)	MLOC_61215 (Chromosome 5H)	Gramine & serotonin pathway	Converts tryptophan to tryptamine (first step in gramine synthesis)	Knockdown reduces gramine accumulation, increasing susceptibility to herbivores	[196]
HvCYP76M8 (*Cytochrome P450*)	MLOC_72190 (Chromosome 3H)	Hordenine biosynthesis	Hydroxylates tyramine to form *N*-methyltyramine (hordenine precursor)	Associated with fungal resistance and allelopathy	[197]
HvAOX1 (*Amine oxidase*)	MLOC_80922 (Chromosome 6H)	Hordenine catabolism	Oxidizes hordenine to “p”-hydroxyphenylacetaldehyde	Regulates hordenine levels in germinating barley	[198]
HvBBE (*Berberine bridge enzyme-like*)	MLOC_40114 (Chromosome 4H)	Benzoxazinoid pathway	Involved in DIBOA (2,4-dihydroxy-1,4-benzoxazin-3-one) biosynthesis	Linked to defense against pathogens and insects	[199]
HvOMT2 (*O-methyltransferase*)	MLOC_50233 (Chromosome 7H)	Avenanthramide-like compounds	Methylates hydroxycinnamoyl derivatives	Possible role in stress-induced phenolic alkaloid production	[200]
HvLDC (*Lysine decarboxylase*)	MLOC_30567 (Chromosome 1H)	Cadaverine-derived alkaloids	Converts lysine to cadaverine (precursor for quinolizidine alkaloids)	Low activity in barley; more prominent in lupins	[201]
